# Online testing for sexually transmitted infections: A whole systems approach to predicting value

**DOI:** 10.1371/journal.pone.0212420

**Published:** 2019-02-22

**Authors:** Katy M. E. Turner, Katharine J. Looker, Jonathan Syred, Adam Zienkiewicz, Paula Baraitser

**Affiliations:** 1 School of Veterinary Sciences, University of Bristol, Bristol, United Kingdom; 2 Bristol Medical School, University of Bristol, Bristol, United Kingdom; 3 School of Population Health and Environmental Sciences, King’s College London, London, United Kingdom; 4 Department of Sexual Health and HIV, King’s College Hospital NHS Foundation Trust, London, United Kingdom; Hofstra University, UNITED STATES

## Abstract

**Background:**

Online testing for sexually transmitted infections has a lower unit cost than testing in clinical services and economic analysis has focused on the cost per test and cost per diagnosis in clinics and online. However, online services generate new demand for testing and shift activity between services, requiring system-level analysis to effectively predict cost-effectiveness.

**Methods and findings:**

Routinely collected, anonymised, retrospective data on sexual health service activity from all specialist services (clinic and online) within an inner London sexual health economy were collated and harmonised to generate a complete dataset of individual level clinic attendances. Clinic activity and diagnoses were coded using nationally standardised codes assigned by clinicians. Costs were taken from locally or regionally agreed sexual health tariffs. *The introduction of online services changed patterns of testing*. *In an inner London sexual health economy*, *online STI testing increased total number of tests*, *the total cost of testing and total diagnoses while slightly reducing the average cost per diagnosis*. *Two years after the introduction of online services 37% of tests in the were provided online and total diagnoses increased*. The positivity of online services is generally lower than that in clinics but varies between contexts. Where the positivity ratio between clinic and online is less than the cost ratio, online services will reduce cost per diagnosis. In this analysis, areas with different classifications as urban and rural had different clinic/online positivity ratios changing the cost effectiveness between areas. Even after the introduction of online services, simple STI testing activity continues in clinics and providers should consider online-first options where clinically appropriate.

**Conclusions:**

Online services for STI testing are not ‘stand alone’. They change STI testing behaviour with impacts on all elements of the sexual health economy. Planning, development and monitoring of such services should reference the dynamic nature of these systems and the role of online services within them.

## Introduction

Sexually transmitted infections (STIs) are of global public health concern, with 357 million new infections of curable STIs (chlamydia, gonorrhoea, syphilis and trichomoniasis) each year [1]. Regular testing for STIs with rapid treatment and partner notification are important strategies for control [2,3] and are traditionally delivered via sexual health clinics, primary care and sexual health outreach. Online ordering of test-kits, sent home for self-sampling, and posted to a laboratory for diagnosis with text message results are increasingly part of the sexual health economy [4–7]. These services increase access to testing [8] and are offered as part of public sector sexual health care in Canada, the United States, Australia, and some European countries including the UK [9–14].

Online testing has a lower unit cost than testing in clinical services [15–16] and may be targeted at lower risk populations [17]. Cost effectiveness analyses to date have focused on the relative costs of service delivery and positivity using cost per diagnosis [15] or cost per test and treatment [16] as the measure of cost effectiveness. However, changes to STI services stimulate changes in testing activity and testing behaviour. This may be the result of user preference for online services [18] or active signposting from clinical services to online ones [19]. Online testing may generate new demand for testing and shift activity between services. These factors may have wider effects on the whole sexual health economy and overall cost-effectiveness of STI testing.

We present a case study analysis and generate models to understand the relationship between these variables and to inform cost effectiveness assessments of similar services in a variety of contexts. The work reported here starts with the cost effectiveness of online services within an inner-city sexual health economy with high rates of sexually transmitted infections. We use this data to generate cost per test and cost per diagnosis at two time points; a) before the introduction of online services and b) after the introduction of free access to online services. We then use this analysis to explore how cost per diagnosis changes in this inner-city area with different combinations of cost and positivity. Finally we provide examples of the same calculations from areas with different rural/urban classifications, using this as a proxy for proximity to specialist sexual health services.

The online service evaluated in this study was accessible at no cost to any resident of the London Boroughs of Lambeth and Southwark and can be viewed at www.sh24.org.uk. Users complete a short online ordering process to provide contact information and essential information on symptoms, recent risk behaviour and safeguarding with a test kit posted within 24 hours in packaging that fits through most letter boxes. Users collect a finger prick blood sample for HIV and syphilis testing. Men collect a urine sample for testing for chlamydia and gonorrhoea and women collect a vulvo-vaginal swab. Men who have sex with men are offered additional rectal and throat swabs. All samples are posted directly to the laboratory by users in pre-paid envelopes with results available by text message (telephone for HIV) within 72 hours. At the time of the study, individuals with a positive result were managed in the same way as asymptomatic clinic attendees and invited to collect their medication at their local sexual health clinic. Patients were followed up at 2 weeks with a telephone call to confirm treatment and were also supported to complete an online partner notification process.

## Methods

### Setting

The primary dataset was collected from two inner London Boroughs (Lambeth and Southwark) with very high rates of sexually transmitted infection (Public Health England, 2016). Analyses were restricted to service use records of residents from each area. Additional summary data were collated from three other areas that use the same online service as examples of different urban/rural areas [20]. The three areas are; Area A (urban with city and town and significant rural areas); Area B (urban with city and town) and Area C (rural with hub towns).

### Data

Routinely collected, anonymised, retrospective data on sexual health service activity from all specialist services within the sexual health economy were collated. This included specialist (i) genito-urinary medicine (GUM) clinics that had focused on the management of sexually transmitted infections and contraception; (ii) community clinics that had focused on the provision of contraceptive services in the past and expanded this to include the diagnosis and management of sexually transmitted infections. Users were assigned a unique ID for all clinic attendances within a single setting. Identifiable data (date of birth and postcode) were converted to year of age and area of residence then removed prior to sharing with the researchers. Each record includes demographic information (unique patient identification number, gender, age at visit, site of visit, ethnicity, area of residence (lower super output area, LSOA code), sexual orientation and clinical information (first / follow-up visit, up to 12 sexual health codes and up to 6 reproductive health codes for service used; and 5 contraception method codes). The study was approved by the 'North of Scotland Research Ethics Committee' IRAS Project ID 169251 with all data manipulation and sharing protocols were approved through [15/NS/0031].

Records of clinic visits for all sexual health attendances in Lambeth and Southwark were collated from January 1st 2014 to 30th September, 2016 from all sexual health service providers. All records were harmonised to generate a complete dataset of individual level clinic attendances (1 record per person per day). Each record includes demographic information (unique patient identification number, gender, age at visit, site of visit, ethnicity, area of residence (lower super output area, LSOA code), sexual orientation and clinical information (first / follow-up visit, up to 12 sexual health codes and up to 6 reproductive health codes for service used; and 5 contraception method codes). Records were excluded from analysis if there were no codes associated with the clinic visit or if individuals were prisoners or aged under 16 years or 100 years or over. (S1 Table). Aggregate routinely collected data on chlamydia positivity in three local authority areas (Area A,B & C) were accessed from Public Health England routinely collected data set from the online service activity database.

### Definition of attendance types

Individual level clinic activity data where an STI test was provided were collated and summarised as “STI test only” (chlamydia, gonorrhoea, HIV, syphilis) or “STI test as part of complex visit” (chlamydia, gonorrhoea, HIV, syphilis plus another service such as treatment or physical examination) (**S1 Table)**. STI testing was separately coded as genital testing only (T4) and the combination of genital, rectal and oral testing (TT) offered to men who have sex with men (MSM). Clinic activity was coded using the Genito-Urinary Medicine Clinical Activity Data Set v2 (GUMCAD) codes, assigned by clinicians during or after the consultation [21] (**S1 Table**). By definition all online activity was a simple STI test as no other services were provided online.

### Positivity

STI diagnosis was extracted based on the GUMCAD codes and positivity was calculated as follows to generate a composite measure of positivity for the four infections studied for online tests and clinic-based tests:

Online test positivity = Number of positive tests (chlamydia, gonorrhoea, syphilis, HIV) / Number of tests (negative + positive)

Clinic test positivity = Number of positive tests +clinical diagnoses (for chlamydia, gonorrhoea, HIV and syphilis) / (Number of negative tests + Number of positive tests + clinical diagnoses)

For clinics we calculated the total number of tests and STI diagnoses during each quarter. Diagnoses in clinics were not necessarily associated with a specific test episode and individuals could be tested in one setting and managed in another, so, to avoid duplication, individuals within each clinic were restricted to one test or new diagnosis (chlamydia, gonorrhoea, syphilis or HIV) per person within a 6 week period (preferentially selecting the record with the most diagnosis codes). These estimates of the total tests and total diagnoses are therefore not exactly equivalent to the online system (where tests and test results are by definition listed concurrently) but they are appropriate for calculating positivity at the system level for this analysis. Treatment codes relating to diagnoses elsewhere were excluded.

Testing and diagnosis codes for syphilis, gonorrhoea, chlamydia and HIV were defined as follows:

STI test: one or more of the following GUMCADv2 codes (T1, T2, T3, T4, T7, T8, TT)

STI diagnosis: one or more of the following GUMCADv2 codes (A1, A2, A3, A4, A5, B(R, O), C4 (R, O), H1, H1a, H1b)

### Costs

Cost for each service were taken from the integrated sexual health service tariff developed by Pathway Analytics on behalf of the London Sexual Health Programme (http://sexualhealthtariff.pathwayanalytics.com/about-the-integrated-tariff). The currency describes the package of care delivered and each currency has two prices known as tariffs:

- A primary tariff–the cost of delivering that care on its own
- An additional tariff–the cost of delivering that care alongside another, more expensive activity.

We assigned the primary tariff for simple STI testing (chlamydia, gonorrhoea, HIV and syphilis) for episodes of care where STI testing was the main activity (£80.58). We assigned the additional tariff (£56.11) for STI testing in episodes of care where other clinical activities were recorded in addition to STI testing. For the online service, we assigned an indicative price of an online service, with the assumption that this is responsive to variation in tariff over time and geography. The cost of testing for men who have sex with men was calculated separately as this high risk group require three tests for chlamydia and gonorrhoea (oral, rectal and urine). The London Sexual Health Tariff cost was used which is the same for primary and additional tariff (£70.24).

### Analysis

The primary analysis focuses on the Inner-London Boroughs, Lambeth and Southwark and on activity that included an STI test. Activity that did not include STI testing was excluded from our analysis. The database was used to evaluate pattern of service use across the whole sexual health economy in Lambeth and Southwark at two time points:

1. Before the establishment of online testing—our baseline,
2. After its introduction

The dates for these different time periods are given in Table 1.

**Table 1:** Dates of the different time periods of analysis.

01/01/2014–31/12/2014	Time period 1: No online service available (baseline)
01/01/2015–31/03/2015	Roll-out and implementation of RCT[j1] on online service (no online data included for this quarter)
01/04/2015–30/06/2016	Time period 2: Online service (SH:24) fully operational in Lambeth and Southwark

We calculated the following:

1. Total number of episodes of ‘STI testing”; the number of episodes of ‘STI testing only’ and the total number of episodes of ‘STI testing as part of a complex visit’ across the whole sexual health economy.
2. Type of service where the STI testing episode occurred (online or clinic)
3. Total number of STI diagnoses and the positivity in each setting (online or clinic)
4. Average cost per test across clinic and online
5. Average cost per positive diagnosis across clinic and online

The key determinants of the cost per diagnosis are the relative cost of online and clinic tests, the proportion of tests which result in a positive diagnosis and the proportion of those testing who are MSM. We undertook additional sensitivity analyses to investigate the effect of

1) changes to the relative costs of online and clinic tests, assuming that the clinic costs remain stable and reflecting changes in the volume and price of online testing;

2) changes in the relative positivity of online and clinic tests reflecting variation in population prevalence or access to sexual health services by geographic area and demography

3) changes in return rate of test kits (this may reflect different populations and/or different levels of support and quality of online self-sampling service)

We then calculated cost per diagnosis in three additional areas with different classifications of rural/urban to look at the impact of context on cost per diagnosis.

## Results

### The impact of online testing on testing activity across the whole sexual health economy

**Fig 1:**
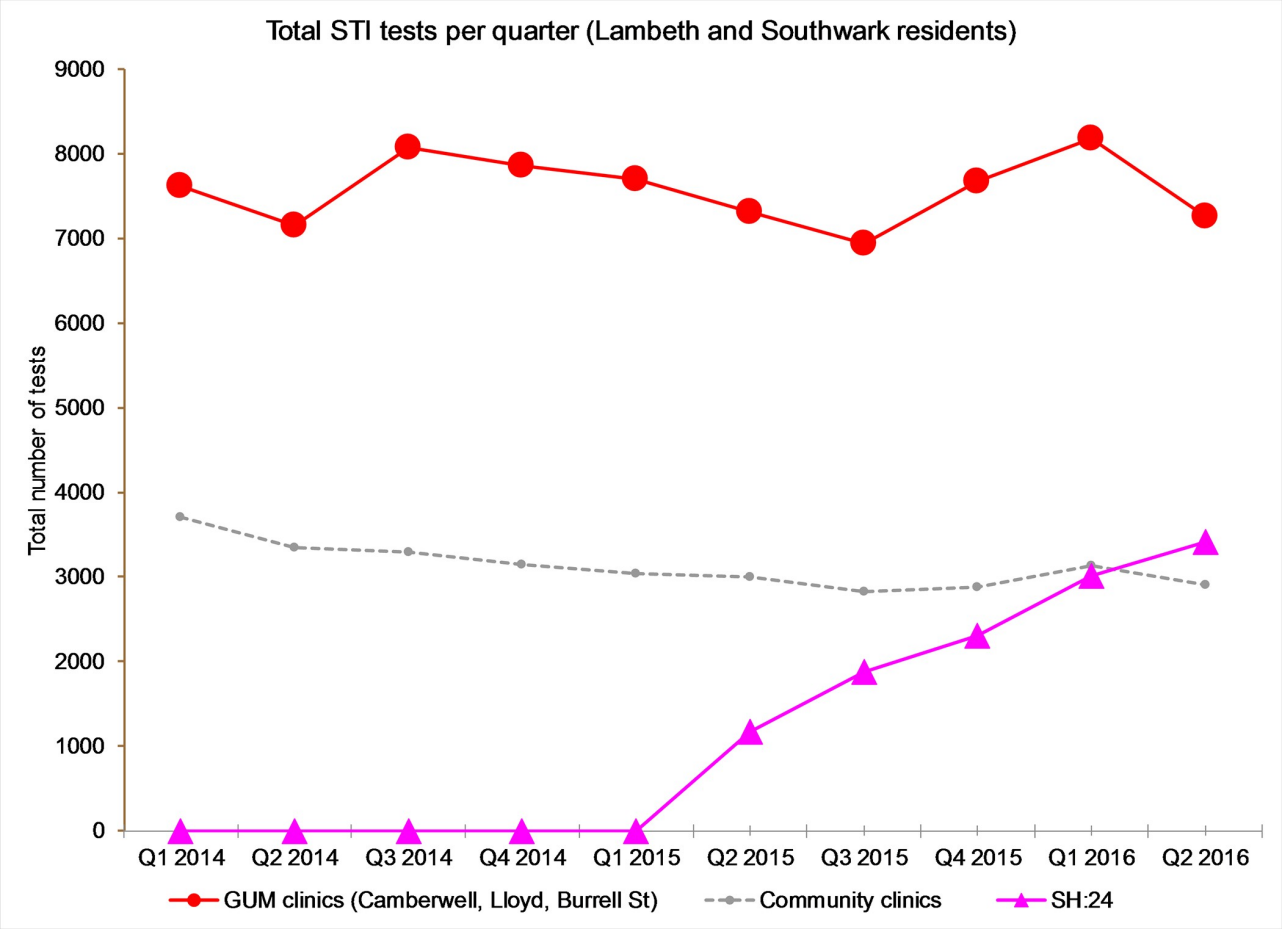
Overall volume of STI testing by service provider and quarter from Q1 2014-Q3 2016.

Testing activity in clinic services remained stable after the introduction of online testing. Testing activity online increased rapidly following its introduction in quarter two, 2015 causing total testing volume across the health system to increase by 27% from 11,003 (mean of quarters 1–3 2014) to 14,027 (mean of quarters 1–3 2016). Tests ordered online as a proportion of total tests across the health system increased to 37% in quarter three 2016. Fig 1 shows STI testing volume in GUM and community clinics and online over the study period.

During the baseline time period (quarter 1–4 2014), STI testing was the primary activity provided in 39% of clinic visits where an STI test was performed and as was an additional activity combined with a more complex clinical service in 61% of visits where an STI test was performed.

### The impact of online testing on positive sti diagnoses across the whole sexual health economy

Positivity is variable quarter to quarter due to variations in factors such as, STI transmission rates and test uptake but the total appears to decrease slightly from Q1 2016 (Table 2). Average positivity within the clinic services is 9.9% (8.8%-11.2%). Average positivity within the online service is 6.7% (5.7% -7.9%). Positivity across the whole system varied between 7.7% and 10.5%.

**Table 2:** STI tests, diagnoses and positivity by quarter split by genitourinary medicine clinic or online (restricted to 1 test or diagnosis per person per 6 week period and to residents of Lambeth and Southwark).

L&S residents, 1 test/diagnosis per person per 6 week*		Q1 2014	Q2 2014	Q3 2014	Q4 2014	Q1 2015	Q2 2015	Q3 2015	Q4 2015	Q1 2016	Q2 2016
Clinics (all)	Total	11150	10337	11168	10843	10483	10187	9603	10405	10977	10024
	Diagnoses	1086	1036	1159	1076	995	1003	1002	1166	1069	948
	Positivity	9.7%	10.0%	10.4%	9.9%	9.5%	9.9%	10.4%	11.2%	9.7%	9.5%
Online	Total	0	0	0	0	0	1165	1883	2298	3008	3414
	Diagnoses	0	0	0	0	0	80	148	169	175	230
	Positivity	.	.	.	.	.	6.9%	7.9%	7.4%	5.8%	6.7%
All	Total diagnoses	1086	1036	1159	1076	995	1083	1150	1335	1244	1178
	Total tests or diagnoses	11141	10349	11163	10839	10485	11355	11483	12711	13980	13434
	Positivity	9.7%	10.0%	10.4%	9.9%	9.5%	9.5%	10.0%	10.5%	8.9%	8.8%

*NB this includes records with an STI test (with or without a positive diagnosis) and any attendances where an STI diagnosis was made without an associated simple STI test, e.g. diagnosis based on i) contact of infection, ii) symptoms, iii) diagnosis elsewhere or iv) microscopy within the clinic.

### The impact of online testing on cost per diagnosis across the whole sexual health economy

Across the whole system, the introduction of online services was associated with changes as detailed in Table 3. There were increases in the average number of diagnoses per month from 363 (period 1) to 399 (period 2) and in the total annual cost of STI testing from £2.87m (2014) to £3.09m (2016). The average cost per test decreased between period 1 and 2 from £66 to £61 and the average cost per diagnosis decreased from £660 to £644. After the introduction of online services, clinics continued to offer simple testing activity as the main clinic activity costing £1,953,652 in the 5 quarters between April 2015 and July 2016.

**Table 3:** Average cost of STI testing before and after the introduction of online testing service.

		Time period 1 Q1—Q4 2014 4 quarters	Time period 2 Q2 2015—Q2 2016 5 quarters
Number of visits	Clinic visit with STI test/diagnosis	43,491	51,191
	Online	0	11,768
Clinic tests	STI test (genital) additional to a complex service requiring a clinic visit	54.9%	49.4%
	STI test (genital, oral, rectal) additional to a complex service requiring a clinic visit	6.5%	3.0%
	STI test (genital) main activity	34.6%	45.8%
	STI test (genital, oral, rectal) main activity	4.0%	1.8%
Costs of tests	STI test (genital) additional to a complex service requiring a clinic visit	£1,339,233	£1,419,751
	STI test (genital, oral, rectal) additional to a complex service requiring a clinic visit	£197,726	£106,976
	STI test (genital) main activity	£1,212,890	£1,887,909
	STI test (genital, oral, rectal) main activity	£123,341	£65,745
	**Total all clinic activity**	**£2,873,191**	**£3,480,380**
	Online tests	£0	£376,576
	**Combined clinic & online costs**	**£2,873,191**	**£3,856,956**
Outcomes	Annual STI test costs	£2,873,191	£3,085,565
	Average STI testing cost per month	£239,433	£257,130
	Average tests per month	3624	4197
	Average diagnoses per month	363	399
	Average cost per test	£66	£61
	Average cost per diagnosis	£660	£644

### Scenario analyses

The key variables which may affect the cost per diagnosis are the relative cost and relative positivity of online and clinic tests, which may vary with; population risk characteristics, testing volume and tariffs.

**Fig 2:**
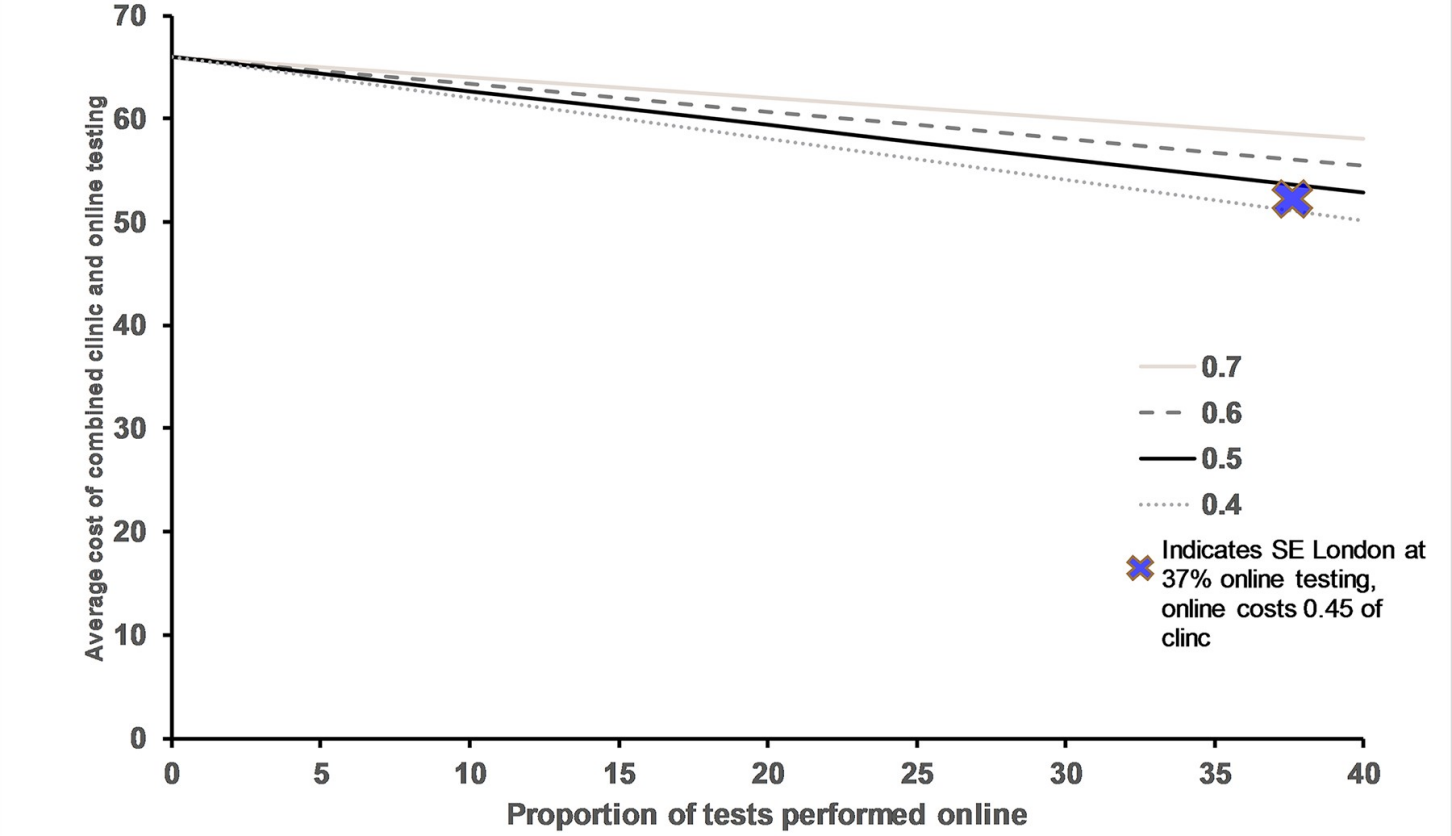
Change in average cost per test (combined online and clinic tests) as the proportion of tests done online increases, under different assumptions of online test costs. Assumptions: Clinic test costs £66. Scenarios: Vary online test cost as a proportion of clinic cost (0.7, 0.6, 0.5, 04).

We considered a hypothetical situation where online cost is 0.7, 0.6, 0.5 or 0.4 times the cost of the clinic service and assumed a linear increase in online testing over time (Fig 2). In our example area, online testing reached 37% as a proportion of total testing with a cost of 45% of the average clinic cost for similar STI testing activity (as the primary reason for attendance) generating a decreased cost per test across the whole system.

**Fig 3:**
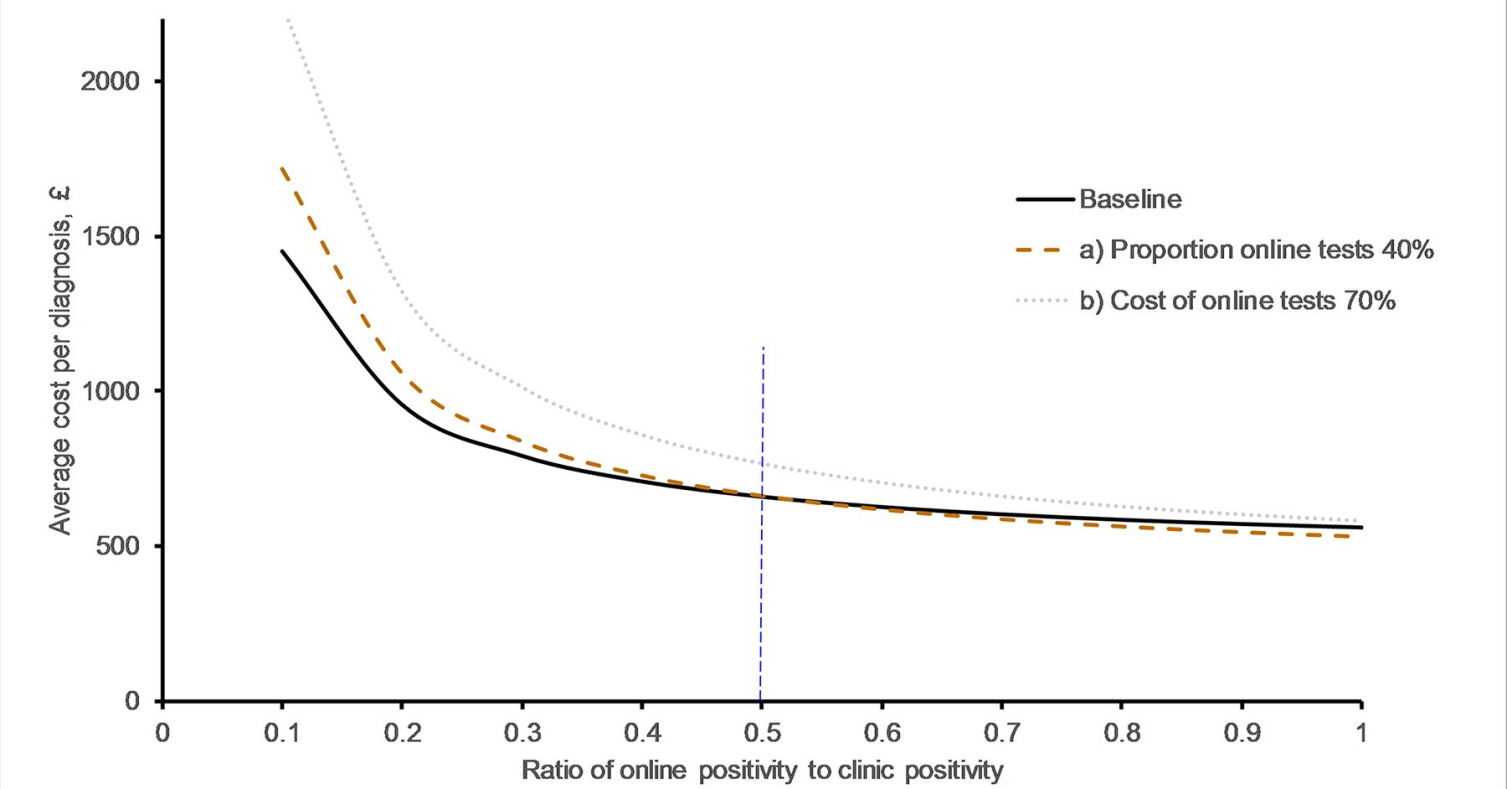
Change in average cost per diagnosis as the ratio of test positivity between clinic and online tests is varied. Assumptions (baseline): 10% of clinic tests are positive, 30% of tests take place online, the cost of online test is 50% of the clinic test cost, the total number of tests is constant. Scenarios: a) Increase the proportion of tests online to 40%, b) Increase the cost of online test to 70% of clinic cost. Red vertical line indicates above online testing decreases the average cost of testing at baseline (positivity ratio 50%).

In addition to cost, positivity influences cost per diagnosis. As the positivity of online tests decreases relative to the positivity of clinic tests the overall cost per positive case within the health system increases, however unless the positivity ratio is lower than the cost ratio, the net effect of increasing online testing will be a reduction in the average cost per positive as shown in Fig 3. If the relative positivity online falls below the relative cost difference then the cost per positive for the online service is higher than for the clinic based service. In our example area the relative positivity is 0.7 and 37% of testing took place online.

**Fig 4:**
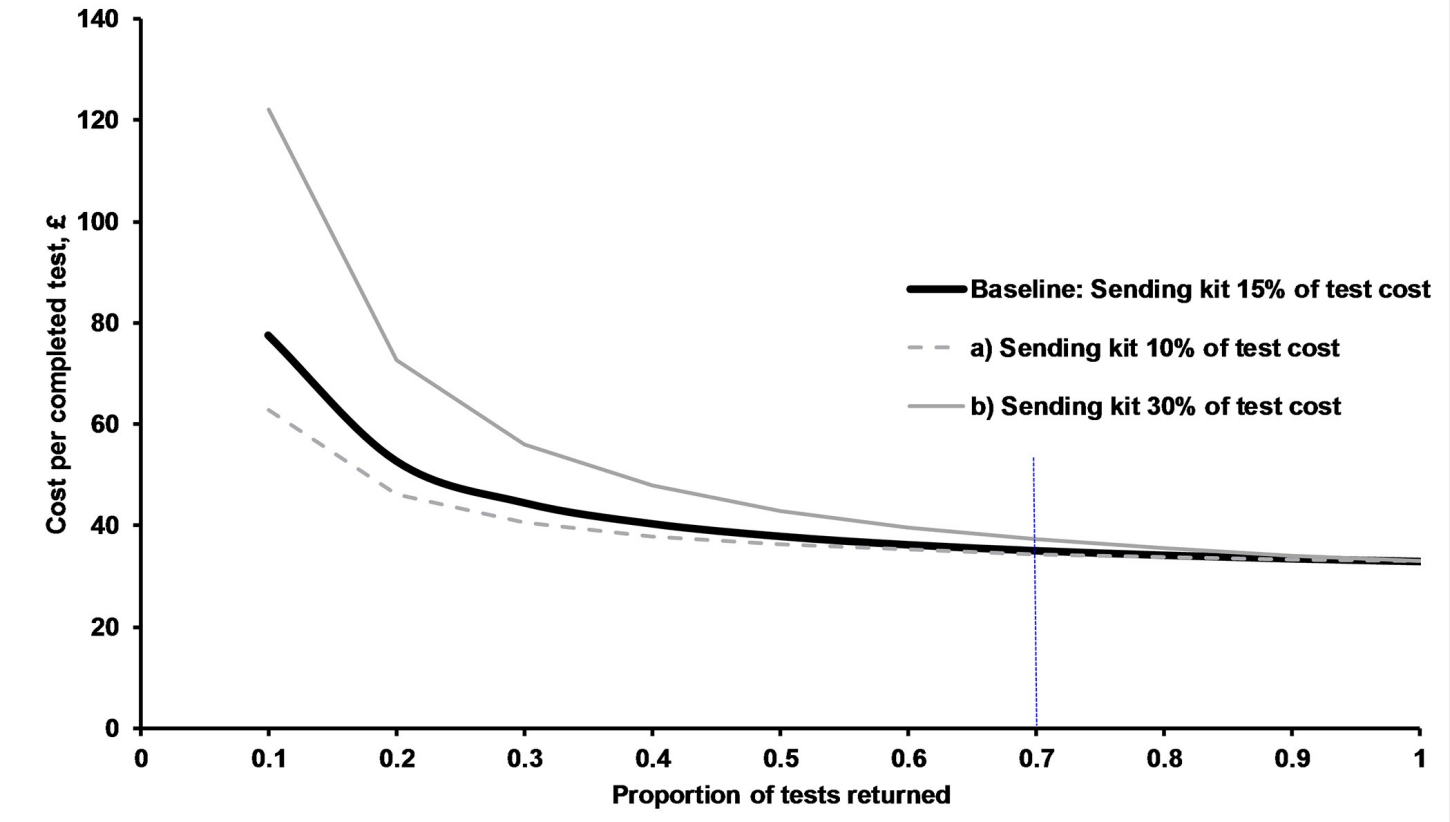
Effect of return rate on average cost per completed test for online test. Assumptions: Online test cost £33, sending out test kit costs 15% of total test cost. Scenarios a) Sending test kit costs 10% of total test cost b) Sending test kit costs 30% of total test cost. Red vertical line: Return rates for the online service in this study were above 70%.

Online services incur costs for test kits which are sent out and not returned. The ‘test ordered cost’, is a small at 15% of the overall cost of providing online testing services and most cost occurs after the kit is returned, for laboratory diagnosis and results management. As the return rate of online tests fall, the cost per completed test increases. The return rate impacts on the cost per completed test once it falls below 60% (Fig 4). The return rate observed in our study was more than 75%.

### Applying this analysis to testing in other areas

As an illustration of the variability of cost per diagnosis with context we conducted a similar analysis for three additional areas in England with different rural/urban classifications. The assumption was that they would have different levels of positivity within the online and clinic service reflecting different levels of STI infection and different barriers to service use. The cost for the online service in each case was the same and we have assumed that the cost of a clinic visit was also the same (£66) although actual clinic budgets and will vary and London tariffs may not apply. This assumption will tend to over-estimate the cost per diagnosis (Table 4) in these settings. On this basis, we estimated cost per diagnosis for chlamydia only in each of the three areas for Quarter 1 2017. Where positivity is similar between clinics and online (as is the case in areas B and C) then the cheaper option (i.e. online testing) offers relatively greater cost benefit.

**Table 4:** Cost per diagnosis in three areas of England using the same online service with SE London as a reference.

	Urban/rural classification	Chlamydia Positivity in clinics	Chlamydia Positivity in online service	Cost per diagnosis in clinic	Cost per diagnosis online
SE London	Urban	7.5%	4.1%	**£880**	**£732**
Area A	Mixed—Urban with cities and towns and significant rural areas	7.9%	5.8%	**£835**	**£517**
Area B	Urban with cities	7.3%	7.2%	**£904**	**£417**
Area C	Rural with hub towns	5.5%	5.5%	**£1,200**	**£545**

Assuming average clinic cost of £66 and online cost 0.45 relative (£30)

Areas A, B and C also commissioned the same online service. We used aggregate routinely collected data on local area clinic chlamydia positivity accessed from Public Health England databases and from the online service activity database over the same time periods as the study.

## Discussion

The introduction of online services changes patterns of testing and therefore cost effectiveness analyses are required at the level of the whole sexual health economy. In areas with unmet need for STI testing, online services are likely to increase total number of tests and total cost of testing but reduce cost per diagnosis as long as the positivity ratio between clinic and online is lower than the cost ratio. Online services can form an important element of sexual health services with 37% of testing provided online in an inner London area two years after the introduction of a free, open-access service for those resident in the area. The relative positivity between clinics and the online service varies between contexts. In our analysis, areas with different classifications of urban and rural had different clinic/online positivity ratios. Where online and clinic positivity is similar then online testing is particularly cost-effective. Even after the introduction of online services, simple STI testing activity continues in clinics and providers should consider online-first options where clinically appropriate.

This paper reports a timely cost-effectiveness evaluation in a rapidly evolving area to provide information to those planning health system innovation. The use of routinely collected data offers consistent information on all attendances at all services in an area and the option of comparisons between areas.

An important weakness of our data set is the lack of information on testing within non-specialist services such as primary care. These services may be particularly important in rural areas where the travel time to a specialist sexual health services may be long. Although there are known barriers to the discussion of sexual health issues with family doctors [22] we acknowledge that simple sexual health testing would be appropriately provided in this context and that our lack of data on this part of the sexual health economy is an important weakness of our analysis. A second weakness of our study is the lack of data on treatment outcomes. In order to provide a truly ‘whole systems’ approach this would have been an important addition to our data.

Changing patterns of disease and rising patient expectations are increasing demands on health services in all health economies [23,24]. UK sexual health services are facing budget reductions [25] and innovations to reduce the costs without compromising quality are required. Supported self-management is one element of the response and can be provided through online health services. Online self-management services work best when integrated with and supported by face-to-face care [26]. This creates interfaces between online and terrestrial services that sustain effective functioning of these emerging hybrid systems. Analysis that acknowledges their interdependence is important to understand their cost-effectiveness. Our findings suggest that policy makers and planners should consider the introduction of online services as one element of the sexual health economy and that these decisions should reflect:

- The unmet need for testing (online services are likely to uncover unmet need)
- The interface between the clinic and the online service, for example ‘digital first’ policies (this changes activity in both settings)
- The populations using both services in terms of risk (positivity) and testing requirements (triple site testing for MSM)
- The test return rate (this will impact on cost per test, particularly if return rates fall below 60%).

These factors are unlikely to remain constant and will require constant review. For, example, new strategies for postal treatment are being developed that will remove the need for those who are tested online to visit clinics at all after a positive diagnosis for chlamydia (although not gonorrhoea which requires an injectable medication). We anticipate that this may increase treatment rates for those tested online. Online testing is increasingly normalised within some populations as clinics are changing their relationship with online services. As testing technologies develop, including a move from self-sampling to self-testing this will impact both the unit cost of tests, the uptake of testing and test return rates [27].

We know of no other studies that use large, routinely collected, data sets to observe the real cost-effectiveness of online testing across the whole sexual health economy in a way that acknowledges the changing testing activity and behaviour stimulated by this service innovation. We know of one study that used randomised controlled trial data to consider the relative cost per test of clinic and online services. This study looked at postal and clinic re-testing after a positive chlamydia diagnosis comparing cost per infection identified ($1409 for postal testing compared to $3133 for cinic based testing) and concluded that online test of cure is cost effective if it shifts activity from clinic to online [28]. We know of two studies that modelled hypothetical cohorts of testers. One [15] predicted a difference in cost per diagnosis from $1281 per STI detected online to $1,593 per STI detected in clinic, by modelling cost of testing and diagnosis for chlamydia, gonorrhoea and trichomonas including the treatment of positives. This study assumed that 67% of those offered an online test would get tested and that 40% of those offered a test in clinic would get tested. It assumed a chlamydia positivity of 8%. This group concluded that online tests were likely to diagnose more infections at a lower cost per test than clinic based services. A second study [16] modelled a hypothetical cohort of 10,000 women per year who received a postal chlamydia screening and concluded that an internet-based screening strategy prevented 35.5 more cases of pelvic inflammatory disease and saved an additional $41,000 in direct medical costs as compared with the clinic-based screening strategy. This assumed a chlamydia positivity of 9.1% and that 35.9% of those offered online tests would take up the offer and complete the testing process and that 26% of women would visit a clinic for chlamydia testing.

Further research is required on the cost effectiveness of online services in a ‘digital first’ system where all users are directed online first and access clinic services only after online testing. Further research is also required to understand the cost effectiveness of online testing in a broader range of contexts including middle income countries where mobile phone use is high.

## Conclusion

Online services for STI testing are not ‘stand alone’. They change STI testing behaviour with impacts on all elements of the sexual health economy. Planning, development and monitoring of such services should reference the dynamic nature of these systems and the role of online services within them. The likely effect of the introduction of online services is an increase in total testing activity, movement of activity between online and clinical services and a decrease in cost per diagnosis as long as he positivity ratio is lower than the cost ratio.

## Supporting information

S1 Table Use of GUMCADv2. (DOCX)

S1 Dataset Data for Fig 2, Fig 3, Fig 4. (XLSX)
