# Supplementary material for: Online testing for sexually transmitted infections: A whole systems approach to predicting value
Source: PLoS One. 2019 Feb 22;14(2):e0212420. doi: 10.1371/journal.pone.0212420 (PMC6386384; doi:10.1371/journal.pone.0212420)
Supplement: S1 Table — (DOCX) [file pone.0212420.s001.docx]

**S1 Table: Use of GUMCADv2 codes to categorise sexual health attendances**

|  | **GUMCAD codes** | | |  |
| --- | --- | --- | --- | --- |
| **Label** | **SHHAPT** | **SHRAD** | **Contraception** | **Description of the codes** |
| *Simple service use  e.g. STI testing / contraception / advice* | | | | |
| **STI test (simple)** | T1, T2, T3, T4, TT, T8, T7, P1A |  |  | Chlamydia, gonorrhoea, syphilis, HIV, Syphilis, self sampling |
| **STI diagnosis** | B (O,R), C4 (O,R), A1, A2, A3, A5, |  |  | STI diagnosis: (Chlamydia, gonorrhoea, syphilis, HIV) |
| **Other** | P3, H, H1, H1B, H1A, D3, PN, PNC, PNG, PNS, P1B, P1C, P3, SW, SRH, T9 | 1 | 5, 6, 7, 8, 12 | Simple contraception  STI treatment diagnosed elsewhere (M suffix)  Advice / health promotion /  partner notification |
| *Complex service use (requiring face-face clinic visit or physical exam/intervention)* | | | | |
| **Complex Services** | A6, T5, T6, T10, TS, C6C, C6B, C7, C1, C3, C15, C13, C14, C10A(M), C10B(M), C2, C12, C16, C4N(R), C5B, D2B, C9, C5A, C8, SG1, SG2, SG3, A7A, C6A, D2A, C11A(M), C11D(M), C2R, C4N(R), C6A, P4A, P4B, P2A, P2B, P2C, P2D, P2E, W1(Q), W2(Q), W3(Q), PEPS, H2, P4, (P2I), PR1, PR2, PR3, 40, 41, PNN, PNT, REF1, REF2, REF3 | 34,19, 20, 21, 22, 23, 35, 36, 29L, 29C, 11, 14, 15, 16,17, 18, 24, 25,26, 37, 38, 39, (29G, 29D), 4, 5, 6, 7, 8, 9, 10, 2, 3, 27, 28, 29, 12, 13, 30, 31, 32, 33, | 1, 2, 3, 4, 9, 10, 11, 13 | Other STI test: HSV, Hep A/B/C, Microscopy  Symptoms/ Diagnosis/ conditions other than above  Other contraception e.g  IUD insertion/removal  Other interventions /vaccination/surgical  Abortion & related  Pregnancy & related  Other services e.g. Sexual assault, referral,pychosexual counselling |
